# A Case of Laryngeal Cryptococcosis that Responded to Itraconazole

**DOI:** 10.1155/2023/8847838

**Published:** 2023-04-12

**Authors:** Sorane Maezumi, Ray Motohashi, Yusuke Shoji, Kiyoaki Tsukahara

**Affiliations:** Department of Otorhinolaryngology, Head and Neck Surgery, Tokyo Medical University, 6-7-1 Nishishinjuku, Shinjuku Ward, Tokyo 164-0023, Japan

## Abstract

Laryngeal cryptococcosis is a rare condition. While there is no reliable evidence regarding the treatment of laryngeal cryptococcosis, oral fluconazole was successful in most previous cases. We experienced a case where we could not continue fluconazole because of adverse drug effects. An 88-year-old female was referred to our department with a 5-month history of sore throat and cough. She had used oral steroids and a corticosteroid inhaler for poorly controlled asthma. Flexible laryngoscopy showed leukoplakia of the vocal cords and subglottic mucosa, and biopsy revealed cryptococcal infection. We started the treatment with fluconazole but changed to itraconazole because of adverse events. Since laryngoscopy performed 6 months later was unremarkable and drug interactions had occurred, we stopped the itraconazole use at 6 months. Our experience suggests that itraconazole is also useful for treating laryngeal cryptococcosis.

## 1. Introduction

*Cryptococcus* spp. is mainly transmitted via the respiratory tract, causing pneumonia and meningitis in many cases [1–3]. Laryngeal cryptococcosis, on the other hand, is a rare condition. In most previous cases, patients were treated with fluconazole (FLCZ) [4]. However, no cases of treatment with alternative drugs have been reported so far. Here, we describe a case of laryngeal cryptococcosis in which the patient was treated with itraconazole (ITCZ) as alternative therapy, because FLCZ had to be discontinued. We obtained the patient's consent to publish this case report.

## 2. Case Presentation

An 88-year-old female was referred to our department with a 5-month history of sore throat and cough. She had been previously diagnosed with brain metastasis from adenocarcinoma of the lung and had been treated with erlotinib. She also had severe asthma requiring oral prednisolone 10 mg and fluticasone furoate/vilanterol inhaler, and tiotropium inhaler. In addition, she had a history of transient ischemic attacks, hypertension, osteoporosis, spinal canal stenosis, right ovarian cyst, endometriosis, and hemorrhoids and was under treatment with montelukast, theophylline, benralizumab, vonoprazan, amlodipine, telmisartan, eplerenone, alendronate sodium, eldecalcitol, suvorexant, eszopiclone, olopatadine, and alprazolam. She was allergic to penicillin and loxoprofen.

**Figure 1:**
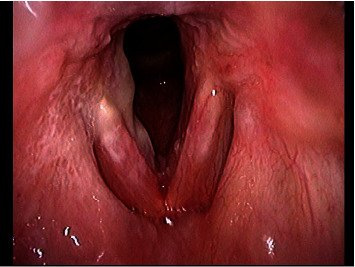
Laryngoscopy findings at the initial examination showing elevated white lesions bilaterally on the vocal cords and subglottis.

**Figure 2:**
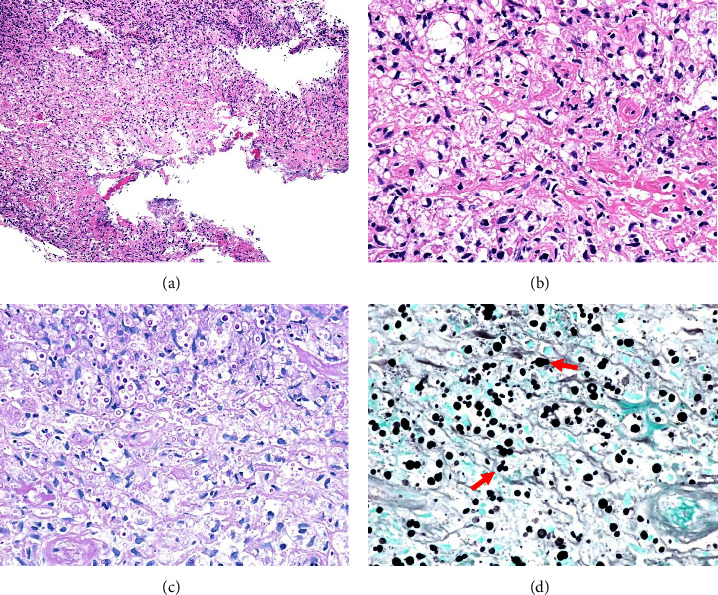
Pathology findings of biopsy specimens. Hematoxylin-eosin staining showed inflammatory cell infiltration and spherical structures (a, b). Yeast-type fungi with capsules that tested positive with PAS and Grocott's staining were observed ((c) PAS staining and (d) Grocott's staining). Budding yeasts (←) were also observed.

Endoscopic examination showed elevated white lesions bilaterally on the patient's vocal cords and subglottic mucosa (Figure 1). Histological examination of a biopsy specimen obtained under local anesthesia at the time of the initial visit showed inflammatory cell infiltration and round fungal bodies (Figures 2(a) and 2(b)). Since these fungal bodies were stained with periodic acid-Schiff (PAS) and Grocott's staining (Figures 2(c) and 2(d)), cryptococcal infection was suspected. In addition, her serum was positive for cryptococcal antigens on latex agglutination. CT scan of the chest revealed no inflammatory changes in the chest.

**Figure 3:**
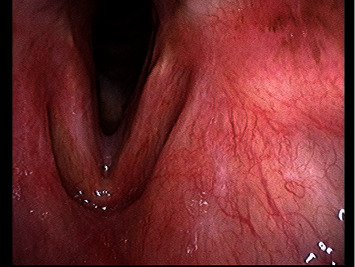
Laryngoscopy findings after 6 months of treatment. The elevated lesions on both sides had disappeared.

Based on the abovementioned part, we diagnosed laryngeal cryptococcosis and consulted a respiratory physician. Considering her advanced age, we started oral FLCZ therapy at a reduced dose of 100 mg/day. However, since she developed pruritus after 1 week, we discontinued FLCZ and commenced treatment with oral ITCZ 150 mg/day. Since there was a risk of weakening of the erlotinib effect due to its interaction with ITCZ, we continued the treatment in coordination with her primary physician, sharing information about the timing of treatment initiation and the risk of weakening of the effect of erlotinib. After 6 months of treatment, the lesions on the vocal cords and subglottic mucosa disappeared bilaterally (Figure 3). Subsequently, based on a report of pleural effusion by the patient's primary physician, we discontinued ITCZ because we thought that the patient's lung adenocarcinoma might have been aggravated by weakening of the effect of erlotinib. At 8 months after the start of treatment, her serum was still positive for cryptococcal antigens, although, since the laryngeal lesions did not recur, we did not resume treatment.

## 3. Discussion

In this case, we started the treatment with FLCZ as given in previous reports but could not continue it beyond 1 week due to adverse events. The subsequent switch from FLCZ to ITCZ resulted in good results. However, because ITCZ interacts with many drugs, it is necessary to understand what other medications the patient might be receiving and to cooperate with the primary physician.

Cryptococcosis is a known opportunistic infection in patients with HIV infection and diabetes mellitus [2]. In cases localized to the larynx, local susceptibility to infection and damage to the mucosal barrier are important predisposing factors, with inhaled steroids being the greatest risk factor [4]. In our case, the patient's asthma had been poorly controlled for several months before onset of the disease, and the combination of inhaled steroids and systemic steroid therapy is likely to have been the main risk factor leading to the development of her cryptococcosis.

There are no established guidelines for the treatment of laryngeal cryptococcosis. When laryngeal cryptococcosis was first reported, it was treated with amphotericin B. However, in 1995, Kerchner et al. reported the first case of successful treatment with FLCZ alone [5]. Since then, many cases that were treated with FLCZ have been reported [4, 6]. The Clinical Practice Guidelines for the Management of Cryptococcal Disease recommend oral FLCZ 400 mg/day for 6–12 months for cryptococcosis other than meningitis and pneumonia [7]. In our case, treatment was started with FLCZ, as in previous reports. However, since the patient developed pruritus after the start of treatment, FLCZ was discontinued. Alternative treatments other than drugs include surgical resection and steroid reduction to reduce risk factors [4, 6], although these alternative treatments were difficult in this patient because of her poorly controlled asthma.

To the best of our knowledge, there are no reports of alternative drug treatments for laryngeal cryptococcosis. The aforementioned guidelines state that oral ITCZ 200 mg/day, oral voriconazole (VRCZ) 200 mg/day, and oral posaconazole (PSCZ) 400 mg/day are acceptable for patients with cryptococcal pneumonia and nonmeningeal cryptococcosis if FLCZ 400 mg is not available [7]. It should be noted, however, that all of these drugs have been reported as a remedy for patients who have been unable to continue treatment with other antifungal agents. In this case, we switched to ITCZ, which is indicated for cryptococcosis in Japan, and her symptoms improved, but ITCZ has one of the highest numbers of drug interactions among antifungal drugs. In the package insert of ITCZ, 25 drugs are listed as being contraindicated with simultaneous ITCZ treatment and many drugs are listed as cautionary drugs, including erlotinib. In this case, the patient was able to complete treatment safely by cooperation between us and her primary physician. However, if further drug switching is required due to drug interactions or side effects, PSCZ and VRCZ should be considered as treatment options, although their use for laryngeal cryptococcosis is off-label in Japan.

## 4. Conclusion

FLCZ is the usual treatment for laryngeal cryptococcosis. In this study, we report a case of successful treatment of laryngeal cryptococcosis with ITCZ as an alternative to FLCZ. If FLCZ cannot be continued due to side effects or drug interactions, we should consider switching to other antifungal agents, including ITCZ.

## Data Availability

Data sharing is not applicable for this case report article.
